# Effect of the Cannabinoid Receptor-1 antagonist SR141716A on human adipocyte inflammatory profile and differentiation

**DOI:** 10.1186/1476-9255-8-33

**Published:** 2011-11-16

**Authors:** Ravi Murumalla, Karima Bencharif, Lydie Gence, Amritendu Bhattacharya, Frank Tallet, Marie-Paule Gonthier, Stefania Petrosino, Vincenzo di Marzo, Maya Cesari, Laurence Hoareau, Régis Roche

**Affiliations:** 1 GEICO, Groupe d'Etude sur l'Inflammation et l'Obésité Chronique, Université de La Réunion, plateforme CYROI, 15 avenue René Cassin, 97715 Saint-Denis Messag Cedex, France; 2 Service de biochimie, Centre Hospitalier Félix Guyon, 97400 Saint-Denis, La Réunion, France; 3 Endocannabinoid Research Group at the Institute of Biomolecular Chemistry of the National Research Council, Pozzuoli (NA), Italy

**Keywords:** human adipocyte, inflammation, SR141716A, TNF-a

## Abstract

**Background:**

Obesity is characterized by inflammation, caused by increase in proinflammatory cytokines, a key factor for the development of insulin resistance. SR141716A, a cannabinoid receptor 1 (CB1) antagonist, shows significant improvement in clinical status of obese/diabetic patients. Therefore, we studied the effect of SR141716A on human adipocyte inflammatory profile and differentiation.

**Methods:**

Adipocytes were obtained from liposuction. Stromal vascular cells were extracted and differentiated into adipocytes. Media and cells were collected for secretory (ELISA) and expression analysis (qPCR). Triglyceride accumulation was observed using oil red-O staining. Cholesterol was assayed by a fluorometric method. 2-AG and anandamide were quantified using isotope dilution LC-MS. TLR-binding experiments have been conducted in HEK-Blue cells.

**Results:**

In LPS-treated mature adipocytes, SR141716A was able to decrease the expression and secretion of TNF-a. This molecule has the same effect in LPS-induced IL-6 secretion, while IL-6 expression is not changed. Concerning MCP-1, the basal level is down-regulated by SR141716A, but not the LPS-induced level. This effect is not caused by a binding of the molecule to TLR4 (LPS receptor). Moreover, SR141716A restored adiponectin secretion to normal levels after LPS treatment. Lastly, no effect of SR141716A was detected on human pre-adipocyte differentiation, although the compound enhanced adiponectin gene expression, but not secretion, in differentiated pre-adipocytes.

**Conclusion:**

We show for the first time that some clinical effects of SR141716A are probably directly related to its anti-inflammatory effect on mature adipocytes. This fact reinforces that adipose tissue is an important target in the development of tools to treat the metabolic syndrome.

## Background

Obesity displays characteristics of a metabolic syndrome, with hyperinsulinemia and resistance to insulin, leading to type II diabetes, atherosclerosis, hypertension, hepatic steatosis, and sometimes cancer [1]. The accumulation of fat in organs and tissues leads to local inflammation, characterized by an increase in pro-inflammatory cytokines such as TNF-a [2]. This is probably one of the decisive steps in the development of insulin-resistance [2]. Obesity is also characterized by the existence of a global inflammatory state, with raised levels of circulating pro-inflammatory cytokines such as TNF-a, C-reactive protein, and IL-6 [3], as well as a reduction in anti-inflammatory cytokines such as adiponectin [4]. Lastly, major modifications of lipid metabolism are also associated with raised circulating triglyceride and fatty acid levels, and with reduction of HDL-C [5].

The development of pharmacological tools is of enormous interest in the fight against obesity and its metabolic consequences. One new physiological pathway of interest is the endocannabinoid system discovered in the early 1990s and believed to influence body weight regulation and cardiometabolic risk factors. This endocannabinoid system consists of two G protein-coupled receptors known as cannabinoid receptors CB1 and CB2; their endogenous ligands, the endocannabinoids, derived from lipid precursors; and the enzymes responsible for ligand biosynthesis and degradation [6,7]. The endocannabinoid system is said to be usually silent and to become transiently activated in stressful conditions. After ligand binding, signalling cascades of cannabinoid receptors can occur through several mechanisms that can act *via *G protein-dependent and independent pathways. Consequently, according to the signalling pathway activated, multiple biological effects are attributed to the endocannabinoid system which has been found to regulate appetite and energy expenditure, insulin sensitivity, as well as glucose and lipid metabolism ([8] for review). Moreover, it seems that the endocannabinoid system exerts many anti-inflammatory actions ([9] for review). Several recent data obtained from studies carried out on animals or humans have demonstrated a close association between obesity and the endocannabinoid system dysregulation, illustrated either by an overproduction of endocannabinoids or by an upregulation of CB1 expression in tissues involved in energy homeostasis ([8] for review). Interest in blocking stimulation of this pathway to aid weight loss and reduce cardiometabolic risk factor development is an area of interest and research. One of the first approaches proposed to reduce the hyperactivity of the endocannabinoid system related to obesity was the development of selective CB1 receptor antagonists such as SR141716A or rimonabant, which has already demonstrated its capacity to improve the clinical picture in obese patients with metabolic disorders. Results from various clinical studies (RIO studies, STRADIVARIUS, SERENADE and ADAGIO) clearly show that treatment with SR141716A leads to weight reduction, an increase in HDL-C levels, a reduction in triglycerides and arterial blood pressure, an improvement in insulin response and glucose uptake, and an increase in adiponectin levels [10-15]. In addition, studies in animal models show that SR141716A is able to reduce the local, hepatic and macrophage levels of pro-inflammatory cytokines [16-18], as effectively as their circulating levels [17,19].

A certain number of clinical effects of SR141716A have been attributed to its direct action on the adipose tissue. This is due to the fact that this tissue is a major player in the development of metabolic disturbances associated with obesity [20], but also because adipocytes express the CB1 receptor and are able to produce and release endocannabinoids [21-23]. Interestingly, it has been postulated that body weight reduction can be linked to inhibition of the cellular proliferation of pre-adipocytes [24] and that the increase in circulating adiponectin is related to increased adipocyte expression of cannabinoid receptors [24,25]. In addition, it has been shown that the treatment of murine pre-adipocytes with SR141716A leads to the inhibition of their differentiation [26], which is in agreement with the finding that CB1 activation instead stimulates pre-adipocyte differentiation [21]. Another recent study demonstrates that a CB1 agonist increases the sensitivity of adipocytes to insulin, whereas SR141716A has the opposite effect [27], which again would agree with the pro-lipogenic role suggested for endocannabinoids acting at CB1 receptors [21]. It is surprising, however, that no studies have been conducted with SR141716A and human adipose cells, which represent the best model to predict the *in vivo *actions of this CB1 antagonist in human white adipose tissue.

Here, we aimed at filling this gap by investigating the effects of SR141716A in human pre-adipocytes and mature adipocytes (exhibiting full fat accumulation) in primary culture. In particular, we have investigated whether the clinical effects of SR141716A have any correlation with the action of this antagonist on human adipose tissue.

## Methods

### Materials

Lipopolysaccharide (LPS from E. *coli *0111:B4 strain, batch #LPE-32-02) was purchased from Sigma (Saint Quentin Fallavier, France). 2-Arachidonoyl glycerol and R1-Methanandamide (2-AG and R1-Met, CB1 agonist, Cayman) were obtained from SpiBio (Massy, France). SR141716 (rimonabant, CB1 antagonist) was a generous gift of SANOFI-SYNTHELABO (Montpellier, France).

### Origin of human adipose tissue samples

Subcutaneous (abdominal, buttocks, hips and thighs) tissue samples of human white fat were obtained from normal weight or slightly overweight human subjects (exclusively females, mean body mass index = 23.3) undergoing liposuction, performed under general anaesthesia, for cosmetic reasons (aged between 25 and 60 years, mean 39 years). Apart from oral contraception, the subjects were not receiving treatment with prescribed medication at the time of liposuction. A total of 21 samples were obtained from 24 patients. The study was approved by the Ile de la Réunion ethics committee for the protection of persons undergoing biomedical research.

### Primary culture of human adipocytes

Cultures were carried out as previously described [22]. Briefly, tissue samples obtained by liposuction were digested for 30 min at 37°C in Ringer-Lactate buffer containing 1.5 mg/mL collagenase (NB5, SERVA, Germany, PZ activity 0.175 U/mg). The floating adipocytes (mature adipocytes) were rinsed three times in Ringer-Lactate. Cells were plated in 24-well (30 000 cells) or 6-well (120 000 cells) tissue culture plates with 199 culture medium supplemented with: 1% Fetal Bovine Serum (FBS) (PAN Biotech, France), amphotericin B, (5 mg/mL), streptomycin (0.2 mg/mL) & penicillin (200 U/mL) (PAN Biotech, France), 66 nM insulin (Umuline Rapide, Lilly, France), 2 g/L glucose, 8 mg/mL biotin and 4 mg/mL pantothenate. Cells were then maintained at 37°C in 5% CO_2 _for a period of 24 hours prior to the experiments.

### Endocannabinoid quantification

Mature adipocytes isolated as described above, were treated or not with 1 μg/ml LPS for 1 or 2 hours. Extraction, purification and quantification of endocannabinoids, 2-AG and anandamide, was achieved as previously described [28]. Briefly, cells with their medium were Dounce-homogenized and total lipids extracted with chloroform/methanol/Tris-HCl 50 mM, pH 7.5 (2:1:1, v/v/v) containing internal deuterated standards (200 pmol [^2^H_5_]-2-AG or [^2^H_8_]-anandamide). After determination of the total lipid content (mg), lipid separation was carried out by using open bed chromatography on silica mini-columns. The pre-purified lipid extracts were then injected on to an HPLC-APCI-MS system (LC2010, Shimadzu, Japan) and compounds identified by single ion monitoring according to the method previously described [28]. Quantification of endocannabinoids was achieved by the isotopic dilution method with amounts expressed as pmol per mg of total lipid extract.

### Purification and differentiation of Stromal Vascular Fraction

Tissue samples obtained by liposuction were digested for 30 min at 37°C in Ringer-Lactate buffer containing 1.5 mg/ml collagenase (NB5, SERVA, Germany, PZ activity 0.175 U/mg). Digested tissue was centrifuged at 900 g for 3 min. The cell pellet (SVF, Stromal Vascular Fraction) harvested after centrifugation was resuspended and incubated twice for 10 min in BLB (blood lysis buffer pH 7, NH4Cl 155 mM, KHCO3 10 mM, Na_2_EDTA 1 mM) to eliminate red blood cells. Cells were then centrifuged at 900 g for 3 min and the pellet was resuspended in ringer lactate and filtered through Steriflip 100 μm (Millipore, France). After centrifugation at 900 g for 3 min, cells were resuspended in 199 medium (PAN Biotech, France). Cell number and viability were assessed by trypan blue dye exclusion.

Around 1 million cells were plated in 60 mm culture flask with Media-1 [M199 + Amphotericin B, (5 mg/mL), streptomycin (0.2 mg/mL) and penicillin (200 U/mL) (PAN Biotech, France), 66 nM insulin (Umuline Rapide, Lilly, France), 2 g/L glucose)] with 20% Fetal Bovine Serum (FBS) (PAN Biotech, France). Cells were then maintained at 37°C in 5% CO_2 _for a period of 24 hours prior to the experiments.

Cells were cultured for proliferation in Media-1 with 10% FBS. After 3 days, cells were treated with differentiating Media-2 [M199 + T3 (1 nM), Cortisol (0.2 μM), Ciglitazone (5 μg/mL), Transferrin (0.1 μg/mL)], without FBS, for 3 days.

Cells were then treated with appropriate concentrations of drugs along with Media-3 [M199 + T3 (1 nM), Cortisol (0.2 μM), biotin (8 μg/L) and pantothenate (4 μg/mL)] for 10 days. Media were changed every 3 days.

After 6 days of differentiation and 10 days of treatment, media samples were collected, and the differentiated adipocytes were scraped from the culture plates using TRIzol reagent for RNA extraction, or wells were assayed for lipid accumulation by oil-red-O staining.

### ELISA assays for TNF-a, IL-6 and MCP-1

Following LPS stimulation for 6 hours, with or without SR141716A, media were assayed for TNF-a, IL-6 content with Ready-SET-Go human ELISA kits (eBioscience, Cliniscience, Montrouge, France), and for MCP-1 content with RayBio human MCP-1 ELISA kit (RayBioTech, Clinisciences, France), according to the manufacturer's instructions. ELISA sensitivity: 4 pg/mL for TNF-a, 2 pg/mL for IL-6 and MCP-1.

### ELISA assay for adiponectin

Mature adipocytes cultured in 24 well culture plates were stimulated with LPS with or without SR141716A for 12 and 24 h. Media were assayed for adiponectin levels by using a commercial Human Adiponectin ELISA kit (RayBiotech, Cliniscience, Montrouge, France). ELISA sensitivity: 10 pg/mL.

### TLR2- and TLR4-binding experiments

HEK-Blue™ LPS Detection Kit and PlasmoTest™ were purchased from Invivogen, France. HEK-Blue-2 and HEK-Blue-4 cells are stably transfected with multiple genes from the TLR2 and TLR4 pathways respectively, and with a reporter gene (secreted alkaline phosphatase) which monitors the TLR binding through NFkappaB activation.

Cells were maintained and plated according to the manufacturers instructions. HEK-Blue-4 cells were then treated with 100 nM and 200 nM SR141716A, with or without 10 ng/mL LPS. Similarly, HEK-Blue-2 cells were treated with 100 nM and 200 nM SR141716A, with or without 1× Positive Control (stock 1000×, provided along with the kit). HEK-Blue-2 and HEK-Blue-4 cells were incubated for 16 and 20 hours respectively, followed by collection of OD values at 640 nm.

### RNA extraction, reverse transcription and real-time quantitative PCR

Cells from 6 well plates (3 × 10^5 ^cells) for mature adipocytes and 60 mm culture plates for Pre -adipocytes were extracted with 500 μL of TRIzol™ reagent (Invitrogen, France). Total RNA was isolated and precipitated according to the manufacturer's instructions. 2 μg of total RNA was reverse-transcribed using random heptamer primers (Eurogentec, Belgium) with MMLV (Invitrogen, France). 1 μl of reverse-transcribed RNA was amplified by PCR on an ABI PRISM 7000 thermal cycler (Applied Biosystems, France) using the Taqman™ Master Mix Kit (Eurogentec, Belgium). The 18S ribosomal RNA (rRNA) gene was used as a reference. Primers and probes sequences of TNF-a, IL-6, A-FABP, Adiponectin and 18S are in Table 1. Quantification of target mRNA was carried out by comparison of the number of cycles required in order to reach the reference and target threshold values (DDCT method). Each analysis reaction was performed in duplicate, with 6 samples per condition.

**Table 1:** Primers and probes sequences

Gene	Primers	Probes
TNF-a	5'-AACATCCAACCTTCCCAAACG-3' 3'-CTCTTAACCCCCGAATCCCAG-5'	5'-FAM-CCCCCTCCTTCAGACACCCTCAACC-TAMRA-3'

IL-6	5'-TCACCTCTTCAGAACGAATTGACA-3' 3'-AGTGCCTCTTTGCTGCTTTCAC-5'	5'-FAM-TACATCCTCGACGGCATCTCAGCCC-TAMRA-3'

18S	5'-CGCCGCTAGAGGTGAAATTCT-3' 3'-CTTTCGTAAACGGTTCTTAC-5'	5'-FAM-ACCGGCGCAAGACGGACCAGA-TAMRA-3'

A-FABP	5'-TGAAAGAAGTAGGAGTGGGCTTTG-3' 3'-ACTAGTAGTCACACTTACCCCT-5'	5'-FAM-AGGAAAGTGGCTGGCATGGCCAA-TAMRA-3'

Adiponectin	5'-TCAATGGCCCCTGCACTACT-3' 3'-CAGGTGGCCTTGAGGAACAG-5'	5'-FAM-CCAACTCCATCTCTAAGTGCCGAACTCATC-TAMRA-3

***Statistical analysis***: Statistical analysis was performed using Microsoft Excel software. Differences were tested for significance by the unpaired Student's t-test. *P < 0,05; **P < 0,005; ***P = ###P < 0.001.

## Results

### Effect of SR141716A on basal- and LPS-induced TNF-a, IL-6, and MCP-1 secretion and gene expression in mature adipocytes

**Figure 1:**
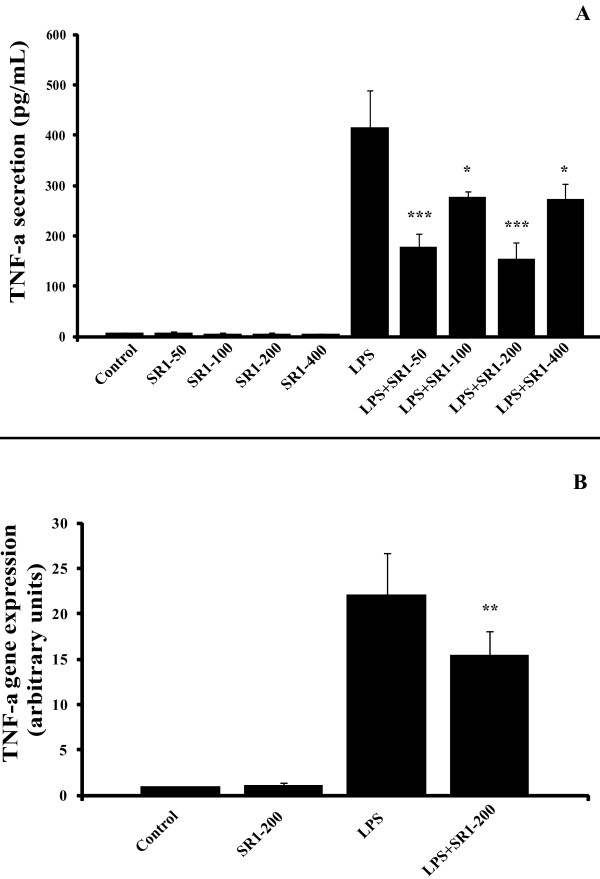
**SR141716A decreases TNF-a secretion and expression in LPS-stimulated mature adipocytes**. Panel A: The concentrations of TNF-a in the medium of mature adipocyte cultures, treated or not with LPS 1 μg/mL alone or in combination with SR141716A were measured at 6 hours by ELISA. SR141716A was used at 50, 100, 200 and 400 nM. Results are expressed in pg/mL. The graph shows the mean ± SD of the results from 3 patients (n = 6 for each condition, for each patient). ***P < 0.001 and *P < 0.05, *versus *LPS-treated cells. Panel B: TNF-a gene expression was determined at 4 hours of treatment in mature adipocyte cultures, treated or not with LPS 1 μg/mL alone or in combination with 200 nM SR141716A. The graph shows the mean ± SD of the results from 2 patients (n = 6 for each condition, for each patient). **P < 0.005, *versus *LPS-treated cells.

**Figure 2:**
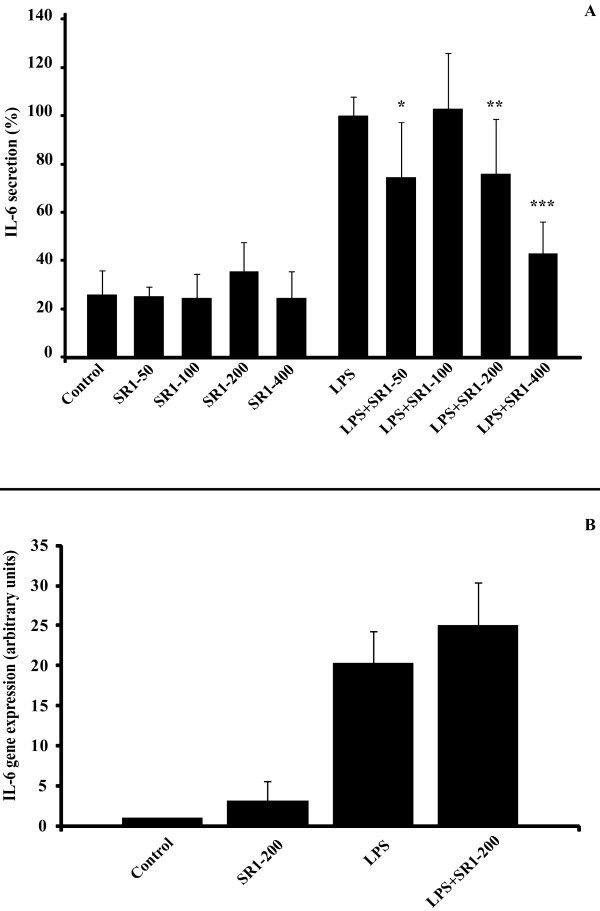
**SR141716A decreases LPS-induced IL-6 secretion but not gene expression in mature adipocytes**. Panel A: Adipocytes were treated with 1 μg/mL LPS and/or not with SR141716A from 50 to 400 nM. IL-6 secretion was measured in media after 12 hours treatment by ELISA. Results are expressed in percentage, normalised to LPS (100% represents from 1 to 10 ng/mL IL6, depending on the patients). The graph shows the mean ± SD of the results from 3 patients (n = 6 for each condition, for each patient). ***P < 0.001, **P < 0.005 and *P < 0.05, *versus *LPS-treated cells. Panel B: IL6 gene expression was determined at 4 hours of treatment in mature adipocyte cultures, treated or not with LPS 1 μg/mL alone or in combination with 200 nM SR141716A. The graph shows the mean ± SD of the results from 2 patients (n = 6 for each condition, for each patient).

It has already been demonstrated that adipocytes express innate immune receptors, such as TLR-4 and TLR-2 and are capable of secreting TNF-a when stimulated with bacterial LPS. When mature adipocytes were treated with LPS (1 μg/mL), the addition of SR141716A at a concentration still selective for CB1 *versus *CB2 receptors (50 to 400 nM) for 6 hours led to a significant decrease in the secretion of TNF-a (around 30%, Figure 1A) and IL6 after 12 hours (around 25%, Figure 2A).

Comparable results were obtained when the expression of TNF-a mRNA was investigated. Co-treatment of adipocytes with LPS (1 μg/mL) + SR141716A (200 nM) brought about a 30% reduction in LPS-induced TNF-a mRNA (Figure 1B). However, we found no significant change in IL-6 gene expression after the co-treatment (Figure 2B).

**Figure 3:**
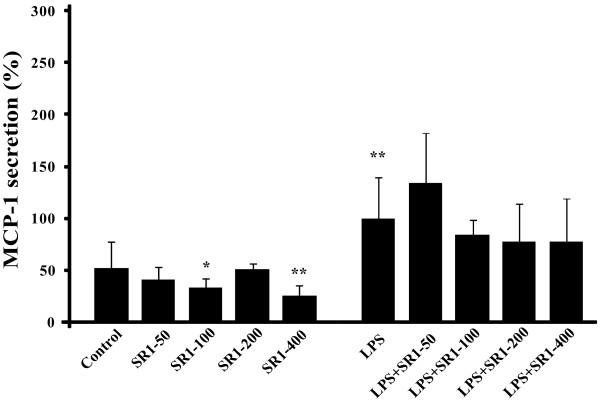
**SR141716A decreases the basal MCP-1 secretion, and slightly the LPS-induced MCP-1 secretion in mature adipocytes**. Adipocytes were treated with 1 μg/mL LPS and/or not with SR141716A from 50 to 400 nM. MCP-1 secretion was measured in media after 6 hours treatment by ELISA. Results are expressed in percentage, normalised to LPS (100% represents from 2 to 5 ng/mL MCP-1, depending on the patients). The graph shows the mean ± SD of the results from 3 patients (n = 6 for each condition, for each patient). **P < 0.005 and *P < 0.05, *versus *control cells.

Concerning MCP-1, SR141716A seems to have an effect on basal secretion, whereas secretion induced by LPS was not significantly affected (Figure 3).

Thus, SR141716A seems to have a broad anti-inflammatory effect on mature human adipocytes, but the mode of action is specific to each cytokine.

### Anti-inflammatory effect of SR141716A is not TLR4-, nor TLR2-dependant

**Figure 4:**
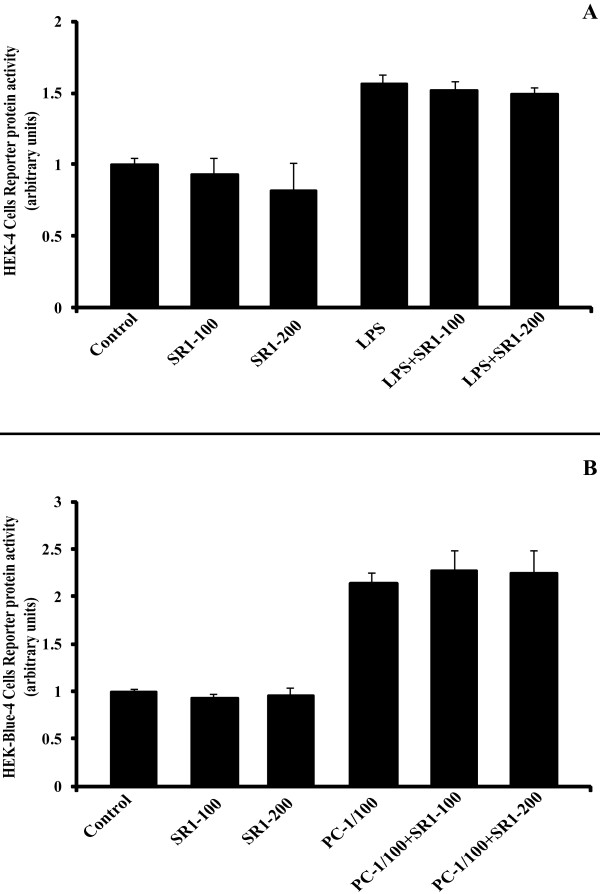
**SR141716A does not bind to TLR4, nor to TLR2**. HEK-4-Blue (panel A) and HEK-2-Blue cells (panel B) were treated with SR141716A (100 nM and 200 nM), with or without their respective positive control: 10 ng/mL LPS and 1/100X Positive Control (PC), for 20 and 16 hours respectively. The graphs show the mean ± SD of the results of 2 experiments (n = 12 for each conditions).

The secretion of TNF-a is mediated by the activation of the NFkappaB pathway, following the binding of LPS to TLR4, with CD14 mediating this effect. In order to find out if the anti-inflammatory effect of SR141716A is due to a TLR4-blocking effect, we treated the HEK4-Blue cells (and the HEK2-Blue cells) with 100 nM and 200 nM of SR141716A, with or not a positive control (10 ng/mL LPS for HEK4-Blue cells and 1X Positive Control for HEK2-Blue cells), for 20 and 16 hours respectively. The Figure 4 shows the reporter protein activity normalized to control cells, which represents NFkappaB activation, and thus TLR-binding. It is quite evident that there is no binding between SR141716A and TLR4 (Figure 4A), nor with TLR2 (Figure 4B).

### LPS induces secretion of the endocannabinoid 2-AG in mature adipocytes

**Figure 5:**
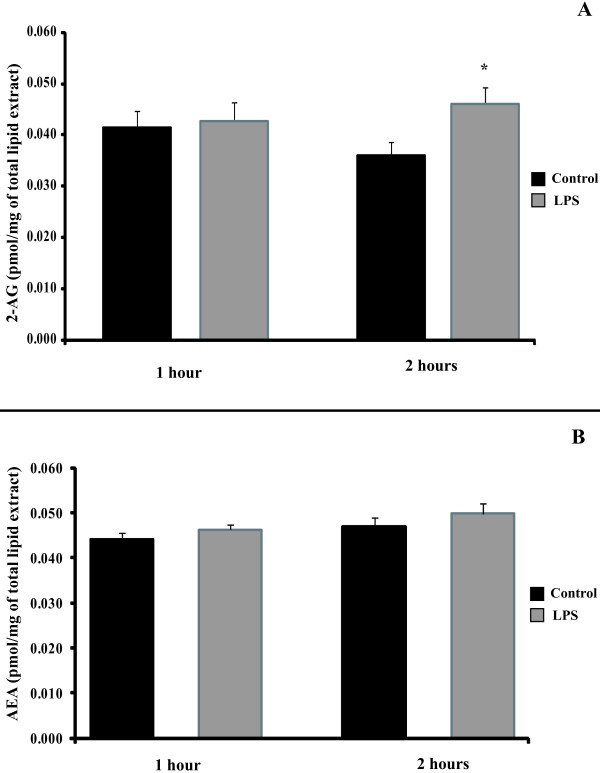
**2-AG, but not anandamide (AEA), is induced by LPS in human mature adipocytes**. Adipocytes were incubated with LPS 1 μg/mL for 1 and 2 hours. Medium and cells were collected and total lipids were extracted. The CB1 agonists 2-AG (panel A) and anandamide (AEA) (panel B) were identified and quantified by HPLC-APCI-MS analysis. The graphs shows the mean ± SD of the results from 3 patients (n = 6 for each condition, for each patient), *P < 0.05, *versus *control cells.

In order to assess whether the effect of SR141716A on TNF-a secretion was due to inverse agonism or to antagonism of tonically active endocannabinoids, we analysed whether or not LPS induces the formation of 2 endocannabinoids, 2-arachidonoyl glycerol (2-AG) and arachidonoyl ethanolamine (AEA or anandamide) in human adipocytes. Over short incubation time intervals (1 and 2 hours), LPS (1 μg/ml) induced the secretion of the classical CB1 agonist, 2-AG, in mature adipocytes (Figure 5A). The maximal effect of LPS was observed at 2 hours post treatment. No effect on the other endocannabinoid, anandamide, was observed (Figure 5B).

### SR141716A restores secretion of adiponectin in LPS-treated mature adipocytes

**Figure 6:**
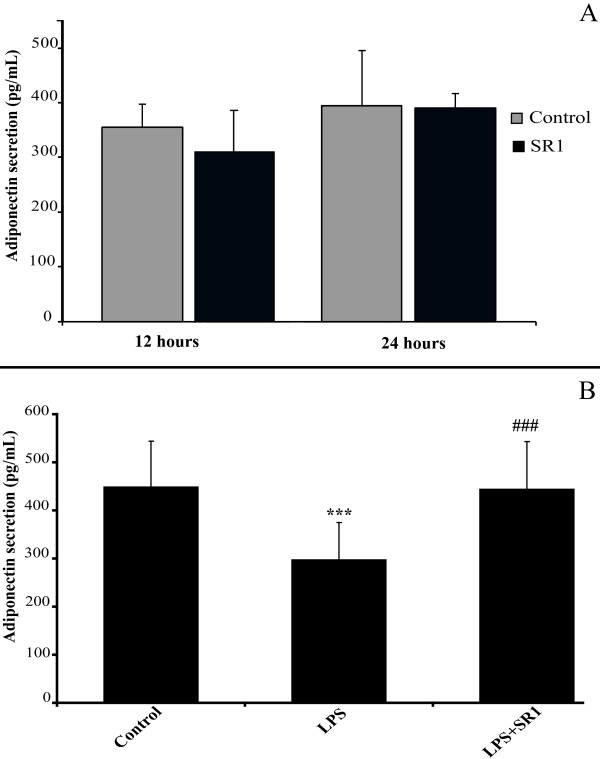
**SR141716A has no effect on basal adiponectin secretion, but restores basal level in LPS-treated mature adipocytes **. Panel A: Mature adipocytes were treated or not with 200 nM SR141716A for 12 and 24 hours. The medium was assayed for adiponectin using ELISA. The graph shows the mean ± SD of the results from 3 patients (n = 6 for each condition, for each patient). Panel B: Mature adipocytes were treated or not with LPS 1 μg/mL, alone or in combination with 200 nM SR141716A for 24 hours. The medium was assayed for adiponectin using ELISA. The graph shows the mean ± SD of the results from 5 patients (n = 6 for each condition, for each patient). ***P < 0.001, *versus *control cells. ###P < 0.001, *versus *LPS-treated cells.

Adiponectin is one of the most important adipokines secreted by adipocytes. It has been previously shown in a murine cell line that SR141716A stimulates adiponectin protein as well as gene expression [25]. We thus decided to study the effect of 200 nM, SR141716A, for 12 and 24 hours, on mature human adipocytes. We did not, however, find any significant change in adiponectin protein secretion in SR141716A-treated adipocytes (Figure 6A).

In order to measure adiponectin secretion in LPS-stimulated mature adipocytes, as well as the effect of SR141716A on these cells, we treated adipocyte cells with 1 μg/mL LPS alone or with SR141716A (200 nM). As shown in Figure 6B, LPS caused a decrease in mature adipocyte adiponectin secretion (approximately 30%), at 24 hours of treatment. In this case, co-treatment with SR141716A and LPS reversed this effect, and restored the adiponectin levels to those of the control cells.

### Effect of SR141716A on pre-adipocyte differentiation and gene expression

In order to understand the effect of the CB1 receptor antagonist SR141716A on the differentiation process and its particular effect on fat accumulation, we differentiated human stromal vascular cells (SVF) into adipocytes and observed the level of differentiation using oil-red-O staining, as well as by measuring the expression of well known differentiation gene markers. Pre-adipocytes were treated with SR141716A at 200 nM and 500 nM for 10 days.

**Figure 7:**
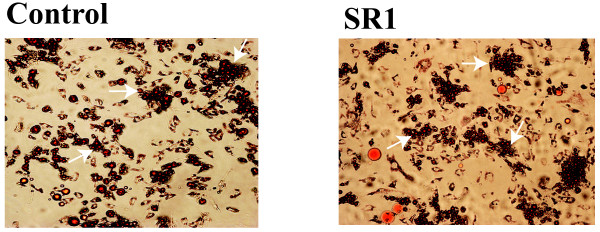
**SR141716A has no effect on oil accumulation in differentiated pre-adipocyte**. Cultures of SVF cells were stained with Oil-red-O after 10 days of SR141716A (200 nM) treatments. Photographs are representative of 3 different experiments on 3 different tissue samples.

**Figure 8:**
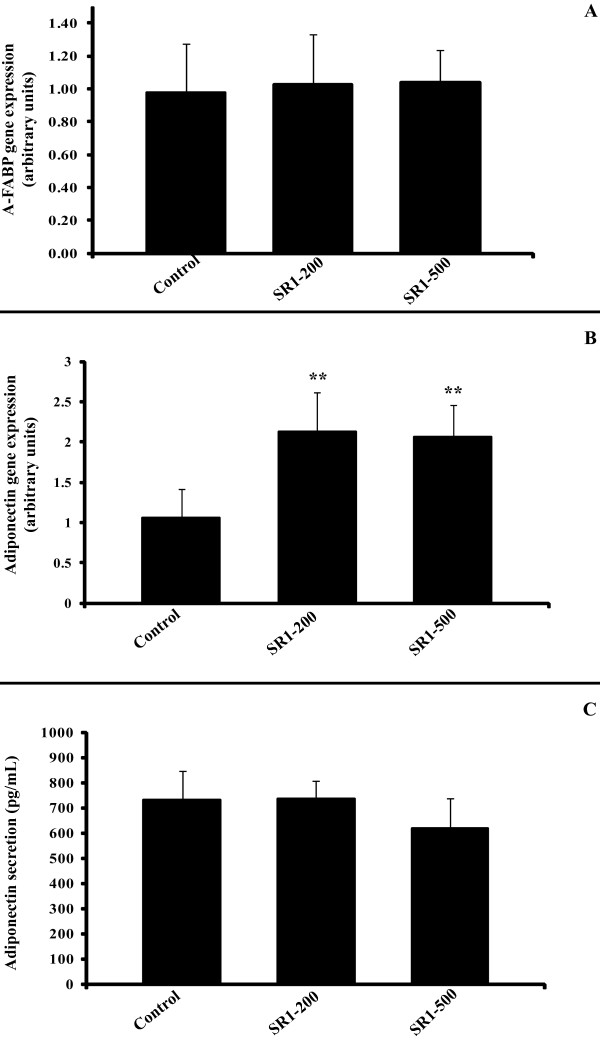
**SR141716A increases adiponectin gene expression, but not secretion, and has no effect on A-FABP gene expression in differentiated pre-adipocytes**. Panel A: A-FABP gene expression was determined at 10 days in differentiated pre-adipocytes cultures, treated or not with SR141716A (200 and 500 nM). The graph shows the mean ± SD of the results from 2 patients (n = 6 for each condition, for each patient). Panel B: Adiponectin gene expression was determined at 10 days in differentiated pre-adipocytes cultures, treated or not with SR141716A (200 and 500 nM). The graph shows the mean ± SD of the results from 3 patients (n = 6 for each condition, for each patient). **P < 0.005, *versus *control cells. Panel C: Differentiated SVF cells were treated or not with SR141716A (100 nM and 200 nM) for 48 hours. The medium was assayed for adiponectin using ELISA. The graph shows the mean ± SD of the results from 2 patients (n = 6 for each condition, for each patient).

SR141716A neither increased nor decreased fat accumulation in these differentiated cells (Figure 7), nor changed the expression of the Adipocyte-Fatty Acid Binding Protein (A-FABP) gene (Figure 8A).

Lastly, we found that treatment with SR141716A led to an increase in adiponectin gene expression (Figure 8B). This effect was significant at 200 nM and 500 nM concentrations. The increase in adiponectin gene expression was not accompanied by an increase in protein secretion as measured by ELISA (Figure 8C).

## Discussion

The adipose tissue is now recognized as being an endocrine tissue capable of secreting a large number of various types of molecules, the adipokines, which are more or less specific to this tissue. Although not an exhaustive list, the following are the main adipokines: leptin, TNF-a, IL-6, adiponectin, MCP1 and IL-10. It has largely been demonstrated that these mediators are implicated in pathologies associated with obesity, in particular those associated with local and global inflammation [4,20,29,30].

Furthermore, the adipose tissue should no longer be considered as a passive, fatty acid storage tissue, since numerous studies now demonstrate that it acts in fact like a transitory reservoir also for circulating cholesterol [31-33]. Reverse cholesterol transport occurs following mobilization of cholesterol and adipocyte apoE by developing HDLs (Bencharif *et al*., 2010, under review). Moreover, the interactions between pro- or anti-inflammatory molecules and cholesterol efflux are currently being investigated [33,34].

Lastly, the adipose tissue is also able to produce endocannabinoids, i.e. mediators acting at cannabinoid CB1 and CB2 receptors (anandamide, 2-AG) and endocannabinoid-like molecules, such as PEA and OEA, which act at PPAR-alpha receptors [22,35]. These mediators display an important paracrine or autocrine pro- or anti-inflammatory actions [29,36], since their receptors are expressed on the surface of adipocytes, and in particular in fully differentiated mature adipocytes [23].

Some of the beneficial clinical effects of the CB1 antagonist, SR141716A, have until recently been attributed both to the peripheral action of the molecule on adipose tissue [24,25,37,38], particularly with regard to weight loss and the increase in circulating adiponectin levels [17,24,25] and to the anti-inflammatory action of the molecule against hepatic steatosis and pro-atherosclerotic processes [16-19]. At least some of these peripheral effects of SR141716A can be explained by an overactivity of CB1 receptors caused by permanently elevated levels of endocannabinoids, anandamide and 2-AG, in the visceral adipose tissue, liver and atherosclerotic plaques, as assessed, *in vitro*, in murine adipocytes and, *in vivo*, in animal models of obesity and atherosclerosis [21,36,39]; see [40] for review.

In this study, we show that SR141716A possesses an anti-inflammatory activity also upon mature human adipocytes in primary culture, consisting of a partial but significant inhibition of LPS-induced expression and secretion of TNF-a (Figure 1A and 1B). This result is in agreement with those of Miranville et al., who showed that SR141716A could decrease the macrophage TNF-a production, resulting in a rescue of insulin signaling in adipocyte (Miranville et al., Obesity, 2010). So, the peripheral anti-inflammatory effect of SR141716A on adipose tissue is first due to the direct action of this molecule on adipocytes, but also to an indirect action on infiltrated macrophages.

It is to be noted that the anti-inflammatory effect, in our adipocyte cellular model, includes IL-6 secretion, but not its gene expression (Figure 2A and 2B). These results are in accordance to those obtained by Sugamura *et al. *[18] who demonstrated, in human macrophages treated with LPS, that SR141716A is able to decrease both TNF-a and IL-6 secretion levels. However, Dol-Gleizes *et al*. showed a decrease in LPS-induced IL6 gene expression [16]. However, to obtain significant results, the authors have used a concentration of SR141716A of 1 μM, which can be considered as notably high, or unselective for this kind of molecule. It is probable that the concentration of 200 nM, which was used in the present study, is more selective for CB1, and this, together with the different cell type used here, could explain the difference between the two sets of results. Moreover, this concentration is in accordance with several previous studies on adipocytes [24].

In order to confirm the broad anti-inflammatory action of SR141716A, we have also checked the LPS-induced MCP-1 section. Although the results are not significant, SR141716A seems to decrease this secretion (Figure 3). The same result was obtained by Dol-Greizes *et al*. on MCP-1 gene expression [16]. Moreover, it should be noted that SR141716A is able to reduce the basal MCP-1 secretion, while it's not the case for the other cytokines secreted. This result is crucial, because it shows that SR141716A could act long before the establishment of chronic inflammation, especially as the deleterious effect of MCP-1 has been demonstrated, notably in the macrophages infiltration process [41].

In low-grade inflammatory status, and in our cellular model, activation of "Toll-like receptor 4" (TLR4) with LPS (linked to the LPS-binding protein) is the primordial step leading to cytokines secretion, *via *the NFkappaB pathway. So, the anti-inflammatory effect of SR141716A could be explained by its binding to TLR4, which could block the receptor and then limit the LPS binding. We verified this hypothesis by using HEK4-Blue cells, which have high expression of TLR4 and all genes downstream, and we have proved here that there is no binding between SR141716A and TLR4, nor with TLR2 (another PAMPs receptor) (Figure 4A and 4B). This anti-inflammatory effect of SR141716A seems to be specific to CB1.

We also report here that LPS induces an increase in the secretion of 2-AG by adipocytes (Figure 5A), but not in anandamide synthesis (Figure 5B). This is in accordance with the fact that 2-AG are expressed at a permanently elevated level in inflammated adipose tissue, whereas it's not the case for anandamide [21]. Moreover, we've tested the effect of 2-AG on LPS-induced TNF-a secretion and we are not able to find any pro-inflammatory effect of 2-AG (data not shown). According to that, we can conclude that the anti-inflammatory effect of SR141716A is not due to the blockade of 2-AG binding. SR141716A has thus its own effect.

Contrary to the results obtained by Bensaid *et al. *[25] and Matias *et al. *[21], in mouse 3T3 adipocytes, we were unable to show that treatment of mature human adipocytes with SR141716A alone results in an increase in the expression or secretion of adiponectin (Figure 6A). It is likely that the choice of the cellular model, and in particular of a different species, is the cause of this discordance. Alternatively, it is possible that the stimulatory effect of SR141716A on adiponectin expression and release from adipocytes is only observed in the visceral adipose tissue, which is characterised by the strongest pro-inflammatory profile during obesity. Indeed, SR141716A, in clinical use, does restore adiponectin levels in abdominally obese patients (ADAGIO-lipids study, [15]), whose levels are low compared to non-obese patients. This effect could potentially be related also to a reduction in the levels of circulating TNF-a, since there exists a well established inverse regulation between these two molecules [42]. We thus verified this hypothesis by measuring the levels of adiponectin secreted when the cells were treated with LPS (with subsequent increase in TNF-a secretion), or with LPS + SR141716A. Indeed, LPS reduced the release of adiponectin from cells, and co-treatment with SR141716A effectively counteracted this effect in this case (Figure 6B). These results support some of the claims made about the peripheral effects of SR141716A, and in particular the effect upon the adipose tissue. However, it is necessary to stress that SR141716A exhibited here no effect upon adiponectin when the cells were in a non-inflammatory state. Moreover, it is also possible that, in a clinical setting, the effect on adiponectinemia is partially related to weight loss [43], or to a reduction in the visceral *vs*. subcutaneous white adipose mass as a result of the lipolytic effect of the molecule [15,44], or also, that the peripheral effect of SR141716A on the adipose tissue concerns cells other than the adipocytes. This last point is supported by evidence showing that the secretion of adiponectin is not specific to adipose cells [45]. It is, therefore, possible that the peripheral effects of SR141716A on adiponectinemia in human obesity are, in the end, a summation of all of these effects. Interestingly, in rodents, the amelioration of glucose intolerance and insulin resistance observed following treatment of mice with high fat diet- or leptin deficiency-induced obesity with SR141716A was, to a large extent, dependent on the presence of adiponectin [46,47].

Lastly, certain authors put forth the hypothesis that the reduction in body weight observed with SR141716A and other CB1 antagonists/inverse agonists could be related to inhibition of pre-adipocyte cellular proliferation [24]. With this in mind we decided to study the effect of SR141716A on adipocyte differentiation. The evaluation of proliferation in cells of the human SVF is complicated by the fact that is difficult to determine the actual proportion of pre-adipocytes in this fraction. In fact, it is difficult to prove conclusively that there exists a specific anti-proliferation effect in pre-adipocytes or on any other cellular type. On the other hand, adipocyte differentiation can easily be observed *via *lipid accumulation.

Our results show that SR141716A has no effect upon human adipocyte differentiation of SVF cells since we did not observe any change in lipid accumulation (Figure 7), nor any variation in the expression of a key gene of adipocyte differentiation: A-FABP (Figure 8A). The expression of CD36, PPARalpha and PPARgamma genes was also investigated but again no effect was detected (data not shown).

Lastly, unlike what we observed before using differentiated adipocytes, SR141716A increased the expression of the adiponectin gene when administered to pre-adipocytes (Figure 8B). Our results agree with the findings of Bensaid *et al. *[25] obtained in a murine cell line. However, the secretion of adiponectin by differentiated adipocytes was not modified in our set-up (Figure 8C).

## Conclusion

In conclusion, the present results lead us to suggest that some of the effects observed during clinical treatment with SR141716A are due, at least in part, to an effect of the molecule on the white adipose tissue, which is now considered as an important target in the development of molecules to treat the metabolic syndrome.

On the other hand, we could not confirm all the observations obtained previously with CB1 antagonists in murine adipocytes [21,25,48], thus indicating that there might be species-differences in the actions of these compounds in the adipose tissues, and emphasizing again the importance of using human cells to predict possible effects *in vivo *of pharmacological and therapeutic tools.

The clinical development of SR141716A was discontinued because of psychiatric side effects, which are inherent to the central action of CB1 antagonists [49]. The present data confirm that it might be worthwhile, in order to limit these side effects, to develop molecules which exert only peripheral effects, such as CB1 antagonists that do not cross the hemato-encephalic barrier. These compounds, by acting directly on the white adipose tissue of obese individuals, might reduce systemic inflammation and hence contribute to counteract atherosclerosis and insulin resistance.

## Competing interests

No potential conflict of interest relevant to this article was reported.

## Authors' contributions

RR and MC conceived of the study and LH and FT participated in its design. MP, SP and VdM carried out the endocannabinoid quantification. RM and LG carried out the primary culture and the ELISA, with the help of AB and KB. RM and LH carried out the gene expression. RR, RM and LH participated in drafting the manuscript. All authors read and approved the final manuscript.
